# Implementation of tuberculosis infection control measures in designated hospitals in Zhejiang Province, China: are we doing enough to prevent nosocomial tuberculosis infections?

**DOI:** 10.1136/bmjopen-2015-010242

**Published:** 2016-03-03

**Authors:** Bin Chen, Min Liu, Hua Gu, Xiaomeng Wang, Wei Qiu, Jian Shen, Jianmin Jiang

**Affiliations:** 1 Department of Tuberculosis Control and Prevention, Zhejiang Provincial Center for Disease Control and Prevention, Hangzhou, Zhejiang, China; 2 School of Nursing, Wenzhou Medical University, Wenzhou, Zhejiang, China; 3 Department of Science Research and Information Management, Zhejiang Provincial Center for Disease Control and Prevention, Hangzhou, Zhejiang, China; 4 Auditory Research Laboratory, State University of New York, Plattsburgh, New York, USA; 5 Department of Geriatrics, Zhejiang Provincial People's Hospital, Hangzhou, Zhejiang, China; 6 Department of Nursing, Zhejiang Medical College, Hangzhou, Zhejiang, China; 7 School of Laboratory Medicine, Wenzhou Medical University, Wenzhou, Zhejiang, China

## Abstract

**Objectives:**

Tuberculosis (TB) infection control measures are very important to prevent nosocomial transmission and protect healthcare workers (HCWs) in hospitals. The TB infection control situation in TB treatment institutions in southeastern China has not been studied previously. Therefore, the aim of this study was to investigate the implementation of TB infection control measures in TB-designated hospitals in Zhejiang Province, China.

**Design:**

Cross-sectional survey using observation and interviews.

**Setting:**

All TB-designated hospitals (n=88) in Zhejiang Province, China in 2014.

**Primary and secondary outcome measures:**

Managerial, administrative, environmental and personal infection control measures were assessed using descriptive analyses and univariate logistic regression analysis.

**Results:**

The TB-designated hospitals treated a median of 3030 outpatients (IQR 764–7094) and 279 patients with confirmed TB (IQR 154–459) annually, and 160 patients with TB (IQR 79–426) were hospitalised in the TB wards. Most infection control measures were performed by the TB-designated hospitals. Measures including regular monitoring of TB infection control in high-risk areas (49%), shortening the wait times (42%), and providing a separate waiting area for patients with suspected TB (46%) were sometimes neglected. N95 respirators were available in 85 (97%) hospitals, although only 44 (50%) hospitals checked that they fit. Hospitals with more TB staff and higher admission rates of patients with TB were more likely to set a dedicated sputum collection area and to conduct annual respirator fit testing.

**Conclusions:**

TB infection control measures were generally implemented by the TB-designated hospitals. Measures including separation of suspected patients, regular monitoring of infection control practices, and regular fit testing of respirators should be strengthened. Infection measures for sputum collection and respirator fit testing should be improved in hospitals with lower admission rates of patients with TB.

**Strengths and limitations of this study**

- This study evaluated the implementation and practice of tuberculosis (TB) infection control measures among all TB-designated hospitals in a provincial region of China.
- All TB-designated hospitals (n=88) in Zhejiang Province, China were investigated in the cross-sectional survey in 2014.
- Our study had limitations. The survey was conducted on-site with the hospital staff; therefore, it is difficult to avoid response or observation bias. The study was limited to the Zhejiang Province of China, and it may not represent the current TB infection control situation in the central or western provinces of China. In addition, owing to the small sample size, we could not conduct multivariate analysis. Therefore, the effects of infection control measures need to be evaluated in a further study.

## Introduction

China, the second most populated country in the world, accounted for 10% of global tuberculosis (TB) cases in 2014.^1^ On the basis of findings of China's fifth national TB epidemiological survey in 2010, the prevalence of active pulmonary TB was 459/100 000 persons >15 years old among the general population, and the multidrug-resistant (MDR) TB rate was 6.8%.^2^ Zhejiang Province is in southeastern China, with a reported active pulmonary TB incidence of 68.86/100 000 persons in 2010, which was lower than the national average of 78/100 000 persons.^3^ However, with a population of over 50 million people, about 30 000 new TB cases are still reported annually in this province.

TB transmission, especially of its MDR and extensively drug-resistant forms, poses a high occupational risk to healthcare workers (HCWs) at health institutions.^4^ ^5^ HCWs are not sufficiently protected from TB infection in healthcare facilities when infection control protocols are not followed completely.^6^ A systematic review of findings from low-income and middle-income countries indicated that the prevalence of latent TB infection (LTBI) among HCWs ranged from 33% to 79%.^7^ A study conducted in 22 health institutions in Beijing, Inner Mongolia and Shanghai, China reported an annual TB prevalence of 664.76/100 000 among HCWs.^8^ A recent retrospective study of 7-year TB surveillance data (2005–2011) among HCWs in Zhejiang Province reported annual TB register rates of 45.2–58.4/100 000 persons, which were higher than that among teachers, who had an equivalent social economic status.^9^

Recent studies performed in resource-limited countries have shown that even relatively simple control measures to prevent TB infection appear to be inadequately implemented.^10–14^ In a study conducted in South Africa, only 11% and 22% of 51 clinics had infection control policies and provided N95 masks, respectively.^13^ Mechanical ventilation and N95 respirators were not available in all TB treatment centres in a study conducted in Henan Province, China,^15^ and only 5 of 22 (23%) healthcare facilities in Beijing, Inner Mongolia and Shanghai separated patients with suspected TB and conducted fit testing for respirators.^16^ Despite these data, the implementation of TB infection control measures among TB treatment institutions has not been systematically studied in a provincial area of China.

Before 2005, TB diagnostic and treatment services were performed in most regions in Zhejiang Province by local Centres for Disease Control and Prevention (CDCs). During the past decade, these tasks were assigned to local hospitals (TB-designated hospitals) by the government to provide better medical services for patients with TB. Currently, TB diagnostic and treatment services are performed by TB-designated hospitals in all regions of the province.^17^ Therefore, it is very important to develop and implement TB infection control measures to protect HCWs in these designated hospitals. However, the extent of implementation of control measures among the TB-designated hospitals in Zhejiang Province is unknown; therefore, we conducted a cross-sectional survey to assess the situation. The study objectives were to understand the implementation and practice of TB infection control measures within these hospitals and to explore factors related to their implementation.

## Method

### Study design and setting

A cross-sectional survey was conducted among TB-designated hospitals in Zhejiang Province. The province consists of 11 prefectures and 90 counties. TB services in Zhejiang Province are provided by provincial-level, prefectural-level and county-level TB-designated hospitals. These three levels were classified according to the administrative system in China. The provincial level is the highest level in the province. Provincial-level hospitals are located in the capital of the province and provide medical service mainly to patients who are critically ill with TB or with MDR-TB. The prefectural level is the second level, providing services for 4–12 counties; prefectural-level TB hospitals provide medical services to patients with MDR-TB or with TB from the administrative area. The county level is the lowest level, and county-level hospitals care for local patients with TB. Zhejiang Province has one provincial-level, 12 prefectural-level and 75 county-level TB-designated hospitals. Each prefecture-level city has a local designated hospital, except for two designated hospitals in Hangzhou, but each county does not have its own TB-designated hospital because the prefectural-level TB-designated hospitals might provide services for some of the governed counties. All of the 88 TB-designated hospitals have outpatient TB clinics. However, only 74 of the TB-designated hospitals have inpatient TB wards. All of the TB-designated hospitals were invited and agreed to participate in the survey.

### Data collection

Data collection was conducted between September and December 2014. A facility-level survey and direct observations were conducted in the 88 TB-designated hospitals. The TB infection control questionnaire was based on the WHO TB infection control policy^5^ and the China TB infection control manual.^18^ The questionnaire included the hospital characteristics (eg, facility level, facility type and number of staff), TB patient load, and implementation and practice of TB infection control measures. These included managerial, administrative, and environmental infection control measures and personal protection practices in 2014. The numbers of outpatients and inpatients with TB were obtained from the annual patient register book for 2013. The annual number of hospital staff was obtained from the hospital's annual statistics data for 2013. The interviews were conducted within 3 days after we notified the investigators. During on-site visits, the investigators, through direct observation, also assessed the location of sputum collection, patient triage, ventilation methods, disinfection methods and use of N95 respirators by HCWs. Among the environmental infection measures, we used multiple choice responses to collect the two ventilation and disinfection methods used most often in the outpatient consulting room, inpatient TB ward, outpatient waiting area and outpatient sputum collection area. Trained CDC staff at the provincial, prefectural and county levels conducted the survey. The researchers trained the investigators to ensure a unified, standard approach. The trained investigators organised the survey in their local area and collected the basic information via interview. All of the questionnaires were checked by the investigators at the prefectural level and were delivered to the researchers at the provincial level. The researchers selected eight hospitals (10%) at the provincial level for a quality check. The data were double-checked for completeness and consistency.

### Statistical analysis

Data analysis was performed using SPSS, V.19 (IBM Corp., Armonk, New York, USA). Descriptive analysis was used to summarise the characteristics of the designated hospitals and infection control implementation. Univariate logistic regression analyses were used to assess the relationships between the characteristics of the TB-designated hospitals and TB infection control practice. Three TB infection control measures which were not implemented well were selected as indicators for factor analysis: whether the hospital had a TB infection control plan, whether the hospital had a dedicated sputum collection area (a well-ventilated area), and whether the hospital conducted fit testing for N95 respirators. Crude ORs and 95% CIs were obtained for each association. A p value <0.05 was considered statistically significant. All of the investigated hospitals agreed to participate in the survey.

## Results

The majority of the designated hospitals (94%) were general hospitals. The median numbers of HCWs in the designated hospitals, TB outpatient clinics and inpatient TB wards were 825, 3 and 18, respectively. Each hospital had a median of three infection control staff. The median annual numbers of outpatients and inpatients with TB in each hospital were 3030 (IQR 764–7094) and 160 (IQR, 79–426), respectively. The median number of outpatients with TB treated per staff member per year in the TB clinics was 1263 (IQR 202–1985). Table 1 summarises the key characteristics of the designated hospitals.

**Table 1:** General characteristics of 88 tuberculosis (TB)-designated hospitals in Zhejiang Province, China, 2013

Variable	Results
Hospital type	
General hospital	83 (94)
Specific hospital	5 (6)
Hospital level	
Provincial	1 (1)
Prefectural	12 (14)
County	75 (85)
Number of staff in each hospital	825 (497–1185)
Number of staff in each TB outpatient clinic	3 (2–6)
Number of staff in each inpatient TB ward	18 (12–23)
Number of infection control staff in each hospital	3 (2–5)
Annual outpatient turnover in each outpatient clinic	3030 (764–7094)
Annual confirmed TB patient turnover in each hospital	279 (154–459)
Annual TB inpatient turnover in each inpatient TB ward	160 (79–426)
Annual number of outpatients per staff member in each TB outpatient clinic	1263 (202–1985)
Annual number of TB inpatients per staff member in each inpatient TB ward	11 (6–24)
Annual number of confirmed TB patients per staff member treated in each TB outpatient clinic	151 (92–252)

Values are reported as n (%) or median (IQR).

### Managerial and administrative control measures

A written TB infection control plan was available in the TB clinic/ward at 72/88 (82%) of the TB-designated hospitals, yet only 51/88 (58%) of the TB-designated hospitals had a TB infection control committee. Most (84/88, 95%) of the TB-designated hospitals reported having a regulation for prompt sputum tests for patients with TB. A referral policy for patients with suspected TB was commonly reported (86/88, 97%). Only 37/88 (42%) of the TB-designated hospitals provided an expedited priority service to shorten the stay for patients with TB, and less than half (46%) had a separate patient waiting area (table 2).

**Table 2:** Implementation of infection control measures in 88 tuberculosis (TB)-designated hospitals

	Implementation	
Infection control measures	N	Per cent
Managerial and administrative infection control		
Establishing TB infection control committee	51	58
Setting a TB infection control regulation or plan in the TB clinic/ward	72	82
Pre-employment infection control training for staff	74	84
Job training on TB infection control measures	60	68
Regulations for regular monitoring of TB infection control in high-risk areas (at least quarterly)*	43	49
Regulation for prompt sputum test for patients with TB	84	95
Rapid referral rules for patients with suspected TB	86	97
Provision of expedited priority service to shorten the stay of patients with TB	37	42
Arranging a separate waiting area for suspected patients	40	46
Health education regarding coughing etiquette and respiratory hygiene for patients	84	95
Environmental infection control		
Dedicated TB outpatient waiting area	68	77
Dedicated sputum collection area (well-ventilated area)†	56	64
Separate entrance for healthcare workers in the TB outpatient clinic	60	68
Separate entrance for healthcare workers in the inpatient TB ward‡	63	85
Regular monitoring of natural and mechanical ventilation (at least quarterly)*	50	57
Daily disinfection of TB outpatient consulting room§	85	97
Daily disinfection of inpatient TB wards§	74	100
Daily disinfection of outpatient waiting room§	83	94
Daily disinfection of sputum collection area§	84	96
Daily disinfection of radiology department§	73	83
Personal respiratory protection		
Supplying TB staff with N95 respirators	85	97
Fit testing on N95 respirators	44	50
Staff screening for TB at least annually	65	74
Offering training on TB infection control	81	92
Annual TB infection control knowledge tests for staff	61	69
Regular monitoring of mask wearing among TB staff (at least quarterly)	75	85

*According to the China TB infection control manual^18^ and China's code for design of infectious diseases hospital,^19^ the infection control measures in the TB clinic should be monitored and meet the requirements in these two regulations.

†A dedicated sputum collection area is where sputum production can be collected in a well-ventilated area.

‡Of all the TB-designated hospitals, only 74 healthcare facilities had inpatient TB wards.

§The disinfection measures by the hospitals complied with the ‘Regulation of disinfection technique in healthcare settings’.^20^

### Environmental control measures

Among all of the TB clinics, 68/88 (77%) had dedicated TB outpatient waiting areas. Fifty-six hospitals (64%) had a dedicated sputum collection area, while 25 hospitals (28%) collected sputum samples in the waiting area, 5 (6%) outside the hospital building and 2 (2%) in the washroom. Table 3 summarises the ventilation and disinfection methods used in the outpatient consulting room, inpatient TB ward, outpatient waiting area and outpatient sputum collection area in the TB-designated hospitals. The majority of the hospitals relied on natural ventilation and ultraviolet germicidal irradiation (UVGI). Mechanical ventilation was used in 30 (34%) outpatient consulting rooms, 23 (26%) outpatient waiting areas, 19 (34%) dedicated sputum collection areas and 20 (27%) TB wards. More than half (50/88, 57%) of the hospitals monitored natural and mechanical ventilation at least once a quarter (table 3).

**Table 3:** Ventilation and disinfection methods in the outpatient consulting room, inpatient tuberculosis (TB) ward, outpatient waiting area and outpatient sputum collection area in TB-designated hospitals

		Outpatient consulting room (N=88)		Outpatient waiting area (N=88)		Dedicated sputum collection area (N=56)*		TB ward (N=74)†	
		N	%	N	%	N	%	N	%
Ventilation method‡									
Natural ventilation	yes	79	90	81	92	51	91	66	89
Mechanical ventilation	yes	30	34	23	26	19	34	20	27
Central air conditioning	yes	19	22	18	20	9	16	22	30
Air cleaner	yes	6	7	2	2	2	4	3	4
Disinfection method‡									
Ultraviolet germicidal irradiation	yes	74	84	68	77	44	79	57	77
Circulating air ultraviolet disinfector	yes	30	34	22	25	15	27	27	37
Electrostatic adsorption type air disinfector	yes	6	7	4	5	3	5	7	10
Chemical disinfection	yes	20	23	18	20	14	25	21	28

*Only 56 healthcare facilities had dedicated outpatient sputum collection areas.

†Only 74 healthcare facilities had a TB ward.

‡Some healthcare facilities used two ventilation and/or disinfection methods.

### Personal protection measures

TB infection control training was available in 81 of the TB-designated hospitals. Most (85/88, 97%) of the hospitals supplied HCWs with N95 respirators, although only 44 (50%) of the hospitals had conducted fit testing for these respirators. Of the 88 TB-designated hospitals, 81 (92%) offered training on TB infection control, 65 (74%) screened staff for TB at least annually, and 61 (69%) tested the infection control knowledge of staff every year.

### Factors associated with practice of infection control measures

In the univariate analysis, no factors were associated with the availability of a TB infection control protocol. Factors associated with a dedicated sputum collection area were staff number and confirmed TB patient load in the TB outpatient clinic. Compared with hospitals with only 1 staff member in the TB outpatient department, those with 3–5 or 6–26 staff members were more likely to have dedicated sputum collection areas (OR=8.66, 95% CI 1.94 to 38.56; OR=7.60, 95% CI 1.60 to 35.90, respectively). Compared with hospitals that had <154 confirmed patients with TB in the TB outpatient clinic annually, those that had 279–458 or 459–6906 patients with confirmed TB annually were more likely to have a dedicated sputum collection area (OR=3.55, 95% CI 0.99 to 12.73; OR=4.80, 95% CI 1.28 to 17.87, respectively). Confirmed TB patient load in the TB outpatient department was associated with fit testing for N95 respirators. Compared with hospitals that had <154 patients with confirmed TB in the TB outpatient clinic annually, those with 279–458 or 459–6906 patients with confirmed TB were more likely to conduct fit testing for N95 respirators (OR=0.19, 95% CI 0.05 to 0.77; OR=0.15, 95% CI 0.03 to 0.59) (table 4).

**Table 4:** Univariate analysis of factors associated with TB infection control measures in designated hospitals

	TB infection control plan			Dedicated sputum collection area			Fit testing on N95 respirators		
	N (%)	OR (95% CI)	p Value	N (%)	OR (95% CI)	p Value	N (%)	OR (95% CI)	p Value
Hospital level									
County level	61/75 (81)	1		46/75 (61)	1		40/75 (53)	1	
Provincial/prefectural	11/13 (85)	1.26 (0.25 to 6.34)	0.77	10/13 (77)	2.10 (0.53 to 8.28)	0.28	3/13 (23)	0.38 (0.11 to 1.37)	0.14
Ranking of per capita GDP in counties*									
Low	32/38 (84)	1		20/38 (53)	1		19/38 (50)	1	
High	29/37 (78)	0.68 (0.21 to 2.19)	0.51	26/37 (70)	2.12 (0.82 to 5.5)	0.11	21/37 (57)	1.31 (0.52 to 3.25)	0.55
Number of staff in designated hospitals									
<497	17/22 (77)	1		11/22 (50)	1		11/22 (50)	1	
497–824	20/22 (91)	2.94 (0.50 to 17.14)	0.23	12/22 (55)	1.20 (0.36 to 3.92)	0.76	10/22 (45)	0.83 (0.25 to 2.72)	0.76
825–1184	18/22 (82)	1.32 (0.30 to 5.77)	0.70	15/22 (68)	2.14 (0.62 to 7.30)	0.22	13/22 (59)	1.44 (0.43 to 4.75)	0.54
1185–4899	17/22 (77)	1.00 (0.24 to 4.09)	1.0	18/22 (82)	4.5 (1.14 to 17.67)	0.31	10/22 (45)	0.83 (0.25 to 2.72)	0.76
Number of staff in the TB outpatient department									
1	10/12 (83)	1		4/12 (33)	1		7/12 (58)	1	
2	14/20 (70)	0.46 (0.07 to 2.80)	0.40	7/20 (35)	1.07 (0.23 to 4.88)	0.92	13/20 (65)	1.32 (0.30 to 5.77)	0.70
3–5	27/32 (84)	1.08 (0.18 to 6.48)	0.93	26/32 (81)	8.66 (1.94 to 38.56)	0.005	14/32 (44)	0.55 (0.14 to 2.12)	0.39
6–26	21/24 (88)	1.40 (0.20 to 9.75)	0.73	19/24 (79)	7.60 (1.60 to 35.90)	0.01	10/24 (42)	0.51 (0.12 to 2.08)	0.34
Confirmed annual TB patient load									
<154	18/21 (86)	1		9/21 (43)	1		17/21 (81)	1	
154–278	18/22 (82)	0.75 (0.14 to 3.84)	0.73	13/22 (59)	1.92 (0.57 to 6.47)	0.28	8/22 (36)	0.13 (0.03 to 0.54)	0.005
279–458	16/22 (73)	0.44 (0.09 to 2.07)	0.30	16/22 (73)	3.55 (0.99 to 12.73)	0.05	10/22 (45)	0.19 (0.05 to 0.77)	0.02
459–6906	20/23 (87)	1.11 (0.19 to 6.22)	0.90	18/23 (78)	4.80 (1.28 to 17.87)	0.01	9/23 (39)	0.15 (0.03 to 0.59)	0.007

*Per capita GDP was ranked according to data from the official website of the Zhejiang Bureau.^21^

GDP, Gross Domestic Product; TB, tuberculosis.

## Discussion

This study evaluated the implementation and practice of TB infection control measures among all TB-designated hospitals in a provincial region of China. According to the previous studies, the common method to assess the effect of TB infection control measures is to investigate the prevalence of LTBI in HCWs.^7^ ^15^ In our study, we used the facility-level interview and observation to reveal the current situations of TB infection control in the hospitals. The findings indicate that most basic TB infection control measures had been undertaken by these TB-designated hospitals in Zhejiang Province. However, they also suggest that some TB infection control measures were not fully implemented in these hospitals.

Managerial and administrative control measures are the first and most important level of control to reduce the exposure of HCWs and other patients to TB.^5^ ^22^ When patients with TB and other facility users share the same crowded and poorly ventilated waiting area, unnecessarily long waiting times in the diagnostic and treatment process can increase nosocomial TB transmission.^12^ ^13^ The aims of managerial and administrative control measures are to ensure rapid diagnosis, isolation and treatment.^23^ Triage and management of patients with suspected TB in outpatient departments are necessary to minimise the exposure of other patients and HCWs,^24^ and the separation of patients with suspected TB is strongly recommended by the WHO.^5^ In countries with a low TB burden, the infection control strategy includes recommendations to isolate patients with TB or MDR-TB from other people in the hospital.^25^ However, this can be difficult in countries with a high TB burden, owing to more patients and fewer resources. In our study, only 46% of the TB-designated hospitals had introduced the segregation of patients with suspected TB. Over half of the TB-designated hospitals provided an expedited priority service to minimise the length of patient stay; longer stays may also increase the risk of nosocomial TB infection.

Environmental control is the second step in reducing the concentration of droplet nuclei in the air.^22^ Ventilation is a vital environmental control measure.^26^ Natural ventilation, such as that through open windows and doors, is efficient and less costly for the movement of air.^27^ Mechanical ventilation is also needed in high-risk areas with poor natural ventilation.^28^ Furthermore, to adhere to the requirements, the effectiveness and function of ventilation should be checked regularly.^19^ ^22^ ^29^ Although natural and mechanical ventilation methods were present in most of the TB-designated hospitals in the present study, the regular monitoring of ventilation (at least quarterly) for TB infection control was conducted in only 50 (57%) of the surveyed hospitals, indicating that many did not sufficiently address this issue, increasing the risk to HCWs. UVGI is also recommended when ventilation is inadequate.^5^ Approximately 80% of the investigated hospitals used UVGI as an environmental infection control measure. In addition, the WHO guidelines recommend that sputum collection should be conducted outside, away from other persons, or in well-ventilated areas.^22^ Unfortunately, this requirement cannot always be met. A study conducted in Uganda indicated that only 42% of healthcare facilities had a designated or well-ventilated area for sputum collection,^10^ and in Mozambique only 20% of the health facilities performed sputum collection in a ventilated outpatient department.^12^ In our study, only 56 hospitals (64%) reported having a dedicated sputum collection area, and 25 (28%) collected samples in the waiting area, which could lead to cross transmission.

Personal respiratory protection is the recommended third and final barrier to protect HCWs from inhaling infectious droplets.^22^ The use of N95 respirators with annual fit testing is effective in preventing nosocomial infections.^30^ Fit testing for respirators is critical to ensure adequate respiratory protection for HCWs^26^ ^31^ and can help staff correctly use respirators and protect the wearer from inhalation hazards.^32^ However, most of the respirators sold in China are designed according to specifications set by a panel of the US Los Alamos National Laboratory, which are based on facial features more typical of adults in Western countries.^33^ Compared with the Western population, Asian populations have higher failure rates in fit testing for respirators of the same size.^34^ In our study, although the use of N95 respirators among HCWs was better than that reported in previous studies,^10^ ^12^ ^13^ only 50% of designated hospitals conducted fit-testing for respirators.

In resource-limited countries with a high prevalence of TB, healthcare facilities cannot effectively implement separation measures due to limited space and budget constraints.^9^ However, in high-income countries with a low TB burden, where the infection control measures are more strict, infection control measures might be neglected by hospitals with low admission rates of patients with TB, owing to limited disease awareness.^25^ As a result, recommended measures are not fully implemented because of scarce resources or less attention to the issue. We found that hospitals with more TB staff (greater than the median number of staff) and a higher patient load (greater than the median patient number) were more likely to have a dedicated sputum collection area and to conduct fit testing for N95 respirators. Higher patient load might encourage hospitals to pay more attention to TB infection control measures. However, fewer admitted patients might reduce the motivation to implement infection control measures, which could lead to nosocomial TB outbreaks.^25^ Since the risk of nosocomial infection exists in all hospitals, infection control measures should also be fully implemented in low-admission hospitals.

## Conclusions

TB infection control measures were generally implemented by the TB-designated hospitals of Zhejiang Province, but the use of some measures needs to be strengthened, including the separation of patients with suspected TB, triage and priority service to shorten the stay of patients with TB, and regularly monitoring infection control and annual fit testing of respirators. Hospitals with lower admission rates of patients with TB should also place more importance on infection control measures such as sputum collection and appropriate respirator usage. Further research methods such as quantitatively measuring the adequacy of ventilation, investigating the prevalence of LTBI in HCWs, and in-depth interview with HCWs could be conducted to assess the effectiveness of TB infection control measures.

## References

[1] WHO. Global tuberculosis report. Geneva: World Health Organization, 2015.

[2] Technical Guidance Group of the Fifth National TB Epidemiological Survey, The Office of the Fifth National TB Epidemiological Survey. The fifth national tuberculosis epidemiological survey in 2010. Chin J Antituber 2012;34:485–508.

[3] Huang Y, Zhong JM, Chen SH Investigation analysis on public awareness of tuberculosis knowledge in Zhejiang province, 2010. Zhonghua Yu Fang Yi Xue Za Zhi 2012;46:352–4. 22800636

[4] Farley JE, Tudor C, Mphahlele M A national infection control evaluation of drug-resistant tuberculosis hospitals in South Africa. Int J Tuberc Lung Dis 2012;16:82–9. 10.5588/ijtld.10.0791 22236851 PMC3875139

[5] WHO. WHO policy on TB infection control in health-care facilities, congregate settings and households. Geneva: World Health Organization, 2009. 24432438

[6] Roth VR, Garrett DO, Laserson KF A multicenter evaluation of tuberculin skin test positivity and conversion among health care workers in Brazilian hospitals. Int J Tuberc Lung Dis 2005;9:1335–42. 16466055

[7] Joshi R, Reingold AL, Menzies D Tuberculosis among health-care workers in low- and middle-income countries: a systematic review. PLoS Med 2006;3:e494 10.1371/journal.pmed.0030494 17194191 PMC1716189

[8] Hou YY, Tan JB, He GX Analysis of the prevalence of tuberculosis disease among healthcare workers in three regions and its associated factors. Chin J Antituberc 2012;6:341–5.

[9] Chen B, Wang X, Zhong J Tuberculosis among healthcare workers in southeastern China: a retrospective study of 7-year surveillance data. Int J Environ Res Public Health 2014;11:12042–52. 10.3390/ijerph111112042 25419877 PMC4245659

[10] Buregyeya E, Nuwaha F, Verver S Implementation of tuberculosis infection control in health facilities in Mukono and Wakiso districts, Uganda. BMC Infect Dis 2013;13:360 10.1186/1471-2334-13-360 23915376 PMC3735480

[11] Reid MJ, Saito S, Nash D Implementation of tuberculosis infection control measures at HIV care and treatment sites in sub-Saharan Africa. Int J Tuberc Lung Dis 2012;16:1605–12. 10.5588/ijtld.12.0033 23131257

[12] Brouwer M, Coelho E, das Dores Mosse C Implementation of tuberculosis infection prevention and control in Mozambican health care facilities. Int J Tuberc Lung Dis 2015;19:44–9. 10.5588/ijtld.14.0337 25519789

[13] Naidoo S, Seevnarain K, Nordstrom DL Tuberculosis infection control in primary health clinics in eThekwini, KwaZulu-Natal, South Africa. Int J Tuberc Lung Dis 2012;16:1600–4. 10.5588/ijtld.12.0041 23032106

[14] Claassens MM, van Schalkwyk C, du Toit E Tuberculosis in healthcare workers and infection control measures at primary healthcare facilities in South Africa. PLoS ONE 2013;8:e76272 10.1371/journal.pone.0076272 24098461 PMC3788748

[15] He GX, van denHof S, van der Werf MJ Infection control and the burden of tuberculosis infection and disease in health care workers in China: a cross-sectional study. BMC Infect Dis 2010;10:313 10.1186/1471-2334-10-313 21029452 PMC2988050

[16] Xiong YC, He GX, Zhao JZ Status of tuberculosis infection control in different levels of healthcare facilities. Chin J Infect Control 2012;4:247–51.

[17] Wu TY, Liu FR Current situation of tuberculosis control in designated hospitals in China. Chin J Antituberc 2014;36:290–3.

[18] Wang LX, Cheng SM, He GX China TB infection control manual. Beijing: Peking Union Medical College Press, 2010.

[19] Ministry of Housing and Urban-Rural Development of the People's Republic of China, General Administration of Quality Supervision Inspection and Quarantine of the People's Republic of China. GB 50849-2014, code for design of infectious diseases hospital. Beijing: China Planning Press, 2015.

[20] Ministry of Health of the People's Republic of China. WS/T367-2012, regulation of disinfection technique in healthcare settings. Beijing: China Standards Press, 2013.

[21] Zhejiang Provincial Bureau of Statistics. Zhejiang Statistical Yearbook 2014 http://www.zj.stats.gov.cn/tjsj/tjnj/ (accessed 28 Jan 2016).

[22] WHO. Guidelines for the prevention of tuberculosis in health care facilities in resource-limited settings. Geneva: World Health Organization, 1999.

[23] Fennelly KP Personal respiratory protection and prevention of occupational tuberculosis. Int J Tuberc Lung Dis 2005;9:476. 15875916

[24] Kompala T, Shenoi SV, Friedland G Transmission of tuberculosis in resource-limited settings. Curr HIV/AIDS Rep 2013;10: 264–72. 10.1007/s11904-013-0164-x 23824469 PMC3809054

[25] Humphreys H Control and prevention of healthcare-associated tuberculosis: the role of respiratory isolation and personal respiratory protection. J Hosp Infect 2007;66:1–5. 10.1016/j.jhin.2007.01.007 17350724

[26] Jensen PA, Lambert LA, Iademarco MF Guidelines for preventing the transmission of Mycobacterium tuberculosis in health-care settings, 2005. MMWR Recomm Rep 2005; 54:1–141. 16382216

[27] Escombe AR, Oeser CC, Gilman RH Natural ventilation for the prevention of airborne contagion. PLoS Med 2007;4:e68 10.1371/journal.pmed.0040068 17326709 PMC1808096

[28] Nardell EA Environmental infection control of tuberculosis. Semin Respir Infect 2003;18:307–19. 10.1053/S0882-0546(03)00069-0 14679478

[29] Standardization Administration of the People's Republic of China, General Administration of Quality Inspection and Quarantine of the People's Republic of China. GB 15982-2012, hygienic standard of disinfection in hospitals. Beijing: China Zhijian Publishing House, Standard Press of China, 2013.

[30] Farley JE, Landers TF, Godfrey C Optimizing the protection of research participants and personnel in HIV-related research where TB is prevalent: practical solutions for improving infection control. J Acquir Immune Defic Syndr 2014;65(Suppl 1):S19–23. 10.1097/QAI.0000000000000035 24321979 PMC3918137

[31] Clayton M, Vaughan N Fit for purpose? The role of fit testing in respiratory protection. Ann Occup Hyg 2005;49:545–8. 10.1093/annhyg/mei046 16148014

[32] Brosseau LM Fit testing respirators for public health medical emergencies. J Occup Environ Hyg 2010;7:628–32. 10.1080/15459624.2010.514782 20853203

[33] Yu Y, Jiang L, Zhuang Z Fitting characteristics of N95 filtering-facepiece respirators used widely in China. PLoS ONE 2014;9:e85299 10.1371/journal.pone.0085299 24465528 PMC3897424

[34] Wilkinson IJ, Pisaniello D, Ahmad J Evaluation of a large-scale quantitative respirator-fit testing program for healthcare workers: survey results. Infect Control Hosp Epidemiol 2010;31:918–25. 10.1086/655460 20658919

